# Residual Angina Should Be Linked to Residual Physiology After PCI

**DOI:** 10.1016/j.jacadv.2026.103390

**Published:** 2026-10-23

**Authors:** Shigetaka Kageyama

**Affiliations:** Department of Cardiology, Shizuoka City Shizuoka Hospital, Shizuoka, Japan

Bouisset et al^1^ elegantly demonstrate that the symptomatic relevance of coronary microvascular dysfunction after percutaneous coronary intervention (PCI) is context dependent: among focal coronary artery disease, coronary microvascular dysfunction was associated with more residual angina, whereas symptoms in diffuse coronary artery disease appeared more related to persistent epicardial burden. We suggest that residual symptoms should be linked to residual, not only baseline, epicardial physiology.

Their interaction analysis uses pre-PCI pull back pressure gradient (PPG), whereas angina is assessed 1 year after revascularization. PCI can alter the spatial distribution of epicardial resistance. Post-PCI fractional flow reserve (FFR) quantifies net residual pressure loss, but a single distal value cannot distinguish focal, diffuse, serial, or mixed residual disease. Diffuse baseline disease is associated with lower post-PCI FFR and less complete functional revascularization.^2^

Crucially, PPG Global repeated post-PCI FFR pull backs, and the parent study reported a pull back-derived residual pressure gradient.^2^ These existing tracings could potentially be reanalyzed alongside baseline microvascular phenotype and Seattle Angina Questionnaire outcomes to ask whether residual symptoms track baseline substrate, residual epicardial pattern, microvascular dysfunction, or their interaction.

Angiography-derived physiology supports this framework. In Multivessel TALENT, diffuse baseline quantitative flow ratio (QFR) pull back pattern predicted suboptimal post-PCI QFR.^3^ Our recently accepted study evaluated post-PCI QFR together with QFR virtual pull back to characterize residual vessel physiology.^4^

Bouisset et al move the field toward integrated epicardial-microvascular phenotyping. Extending the phenotype across PCI may sharpen the mechanistic interpretation: pre-PCI PPG identifies the disease being treated; post-PCI pull back identifies what remains.
